# Changes in beta diversity and species functional traits differ between saplings and mature trees in an old‐growth forest

**DOI:** 10.1002/ece3.6913

**Published:** 2020-12-18

**Authors:** David Anthony Kirk, Marie‐Hélène Brice, Michael S. Bradstreet, Ken A. Elliott

**Affiliations:** ^1^ Aquila Conservation & Environment Consulting Ottawa ON Canada; ^2^ Département de Sciences Biologiques Université de Montréal Montréal QC Canada; ^3^ Québec Centre for Biodiversity Sciences McGill University Montréal QC Canada; ^4^ Nature Conservancy of Canada Toronto ON Canada; ^5^ Divisional Support Section Integration Branch Ontario Ministry of Natural Resources and Forestry Peterborough ON Canada

**Keywords:** Carolinian forest, community traits, homogenization, old‐growth forests, Ontario, protected areas, species at risk, temporal change, β‐diversity

## Abstract

Invasion by generalist tree species can cause biotic homogenization, and such community impoverishment is likely more important in rare forest types. We quantified changes in tree diversity within Carolinian (range in Central Hardwood Forest), central (range in Central Hardwood Forest and Northern Hardwood‐Conifer Forest), and northern species [range reached Northern‐Conifer‐Hardwood/closed Boreal (spruce‐Fir) Forest] in an old forest tract in southern Canada at points surveyed 24 years apart. We asked: How did mature tree and sapling composition and abundance change for the three species’ groups? Did those changes lead to biotic homogenization? Can species’ changes be explained by community traits? We tested for differences in temporal and spatial tree β‐diversity, as well as forest composition and structure, using univariate/multivariate analyses and a community trait‐based approach to identify drivers of change. Major increases occurred in abundance for mature *Acer rubrum* (northern), while other species decreased (*Fraxinus americana*, *Populus grandidentata*); declines were found in *A. saccharinum* (central) and *Cornus florida* (Carolinian). Species composition of saplings, but not mature trees, changed due to replacement; no evidence for biotic homogenization existed in either cohort. As a group, northern mature tree species increased significantly, while central species decreased; saplings of pooled Carolinian species also declined. Shade tolerance in mature trees increased, reflecting successional changes, while drought tolerance decreased, perhaps due to changing temperatures, altered precipitation or ground water levels. Saplings showed declines in all traits, probably because of compositional change. Our results demonstrated that saplings can more closely reflect change in forest dynamics than mature trees, especially over short time periods. Based on sapling trends, this remnant could ultimately transition to a mesophytic hardwood stand dominated by *A. rubrum* and other shade‐tolerant species, creating a more homogeneous forest. While encouraging regeneration for Carolinian and central tree species could ensure high levels of diversity are conserved in the future, it is important to balance this with the primary management goal of maintaining the forest's old‐growth characteristics.

## Graphical abstract

Our results highlight the importance of monitoring tree β‐diversity in long‐term forest plots to examine changes in homogenization, species composition, and functional traits which can lead to management and conservation outcomes. We demonstrated compositional and functional changes in an old growth forest driven mainly by invasion of red maple. Although we did not observe biotic homogenization, these changes indicated potential for future homogenization of this remnant and those of surrounding forests. This raises important conservation issues: whether or not to manage this and other forests to maintain tree biodiversity.

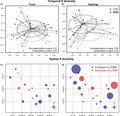

## 1 INTRODUCTION

Biotic homogenization, the process by which the compositional similarity of formerly distinct plant communities and traits gradually increases over time, is escalating in many areas (Clavel, Julliard, & Devictor, 2011; Lôbo, Leão, Melo, Santos, & Tabarelli, 2011; Naaf & Wulf, 2010). While this homogenization has often been associated with the invasion of expanding alien generalist species at the expense of native specialists (Olden, Poff, & McKinney, 2006), it can also occur through the spread of generalist native species (Beauvais, Pellerin, & Lavoie, 2016; Keith, Newton, Morecroft, Bealey, & Bullock, 2009; McCune & Vellend, 2013). For example, the increasing abundance of the generalist tree species *Acer rubrum* in northeastern North America is believed to be contributing to biotic homogenization in some plant communities (Abrams, 1999; Fei & Steiner, 2009; McDonald, Peet, & Urban, 2002; McEwan, Dyer, & Pederson, 2011). Moreover, loss of rare specialist species can also lead to decreasing beta diversity (hereafter β‐diversity) and thus homogenize communities (Olden & Poff, 2002). Such homogenization can detrimentally impact the multi‐functional properties and services of forests (Gamfeldt et al., 2013; van der Plas et al., 2016), although the relationship between high diversity and ecosystem function is equivocal (Ratcliffe et al., 2017).

Temperate forests in fragmented anthropogenic landscapes may be susceptible to homogenization, as many disturbances act individually or synergistically to modify tree community composition (Sonnier, Johnson, Amatangelo, Rogers, & Waller, 2014). In the lower Great Lakes region of southern Canada [called the “Carolinian Zone” or Deciduous Forest Region—equivalent to part of the Central Hardwood Forest of Fralish and Franklin (2002) and the Beech‐Maple‐Basswood Region of Dyer (2006)], the remnant forests scattered throughout anthropogenic landscapes are valued ecologically for their unique and diverse forest communities. This is a result of the mix of northern (e.g., *Tsuga canadensis*, *Pinus strobus*, and *Betula alleghaniensis*) and southern tree species (e.g., *Nyssa sylvatica*, *Liriodendron tulipifera*, and *Sassafras albidum*; Larson, Riley, Snell, & Godschalk, 1999; Waldron, 2003). Although forests containing mixes of northern and southern species are common elsewhere in eastern Canada and the northern United States (e.g., Great Lakes‐St. Lawrence and boreal transitional forest; Danneyrolles, Arseneault, & Bergeron , 2016, 2018), the specific north‐south mix of tree species in the Carolinian Zone is extremely rare in Canada, occurring only in southern Ontario. However, it is widespread in the United States south of the Great Lakes, including the central and southern Appalachians and as far south as North Carolina and other southern states. Because of their uniqueness in our study region (southern Ontario), the loss or gain of these Carolinian species is likely to play a key role in spatial and temporal β‐diversity patterns. While climate warming could favor these warm‐adapted Carolinian species in northern forests (Desprez, Iannone, Yang, Oswalt, & Fey, 2014), the absence of disturbance could impede regeneration as these species are mostly intolerant to shade. Thus, on the one hand, northward expansion of Carolinian species with climate warming could lead to diversification of northern forests. On the other hand, many rare Carolinian species are shade‐intolerant and, in the absence of appropriate natural disturbance within their current range are unlikely to regenerate and establish as canopy trees at new or existing sites, which is a requisite of migration in response to climate change (Davis & Shaw, 2001). Indeed, it has been predicted that many tree species will be unable to respond sufficiently rapidly to adapt to a changing climate (Sittaro, Paquette, Messier, & Nock, 2017; Solarik. Cazelles, Messier, Bergeron, & Gravel, 2020). With successional changes and lack of disturbances such as ground fires and other natural processes altered by humans, rare Carolinian species are likely to decline as shade‐tolerant generalists (e.g., *Acer* species) become predominant, thus potentially leading to homogenization. This trend of *Acer* species succeeding and overtaking shade‐intolerant tree species (e.g., some *Quercus* species) is a widely documented phenomenon in eastern North America (Fei & Steiner, 2007; McEwan, Dyer, & Pederson, 2011; McShea & Healy, 2002; Rogers, Rooney, Olson, & Waller, 2008).

Because the above environmental drivers have opposing influences, predictions about trends in β‐diversity can be challenging (Becker‐Scarpitta, Vissault, & Vellend, 2019; Chapman & McEwan, 2016). In the absence of information on which drivers are most influential, the examination of temporal changes in tree functional traits can provide useful insights (Aubin et al., 2016; Moore, Zhu, Huntingford, & Cox, 2018; Mouillot, Graham, Villeger, Mason, & Bellwood, 2013), including clues regarding changes in functional homogenization, that is, a decrease in functional β‐diversity over time (Sonnier et al., 2014). For example, isolation of forest fragments in anthropogenic landscapes should promote species with long dispersal potential (e.g., in relation to seed size), so trees with strong dispersal traits (e.g., those with animal or wind‐borne seeds) may be over‐represented in more isolated forests compared to weak dispersers (gravity or arthropod‐dispersed; Vellend et al., 2007). As different environmental processes, including pathogens and diseases (Fischer, Marshall, & Camp, 2013), can modify trait composition in forest communities, understanding how these processes drive compositional change is a necessary step toward better informed conservation strategies.

Given the multiple threats to forests in the Anthropocene (Lindner et al., 2010), it is of great importance to monitor changes in tree species size, abundance, and composition in forests (e.g., Knapp & Pallardy, 2018; Legendre & Condit, 2019; McCarthy, Small, & Rubino, 2001; Pinheiro, Goebel, & Hix, 2008; Runkle, 2013; Savage & Vellend, 2015). For example, monitoring of permanent and semipermanent forest plots (“legacy studies”) has provided new insights into linking temporal β‐diversity with climate change and other threats, as well as into the conservation of forest diversity (Becker‐Scarpitta et al., 2019). Moreover, to map and understand global changes in biodiversity, it is necessary to investigate change at specific locales (Blowes et al., 2019). In this paper, we examine resurveyed trees around permanent grid points, 24 years apart, to determine changes in forest structure and composition in a remnant old‐growth forest tract in the Carolinian Zone of southern Ontario, Canada. Because trees are long‐lived and thus forests change very slowly over time, we conducted separate analyses of both mature individuals and saplings, since recent studies have demonstrated that juvenile life stages (saplings and seedlings) may more accurately reflect environmental change (Kribel, Kolman, & Ware, 2011; Mathys, Coops, Simard, Waring, & Aitken, 2018; Sittaro et al., 2017). Specifically, we ask: (a) Are changes in the abundance and composition of tree species over the last two decades related to size distributions of trees (e.g., mature vs. sapling cohorts)? And have trees at the northern edge of their continental ranges in Canada (Carolinian species) become less abundant compared to other tree species that are more broadly distributed in Ontario? (b) Is there evidence for homogenization of tree species composition either for mature tree or sapling cohorts? And (c) Which functional traits of mature trees and saplings have changed over time, and how does this inform us about the processes that are driving these changes? The reduction in natural disturbance rates and patterns due to fire suppression, and the fragmented landscape, has led us to predict that some generalist shade‐tolerant species, such as *A. rubrum*, would increase in abundance at the expense of shade‐intolerant Carolinian species. Furthermore, we expected trees affected by pathogens or diseases to decline (e.g., *Castanea dentata*, *Cornus florida*, *Fraxinus* spp.), regardless of the guild to which they belonged. As a consequence of these compositional changes, we expected to find that the forest has become more homogenized over time (e.g., an increase in generalists—winners, versus a decline in specialists—losers; Wiegmann & Waller, 2006). Finally, we predicted that the observed temporal species compositional changes would be paralleled by changes in functional traits related to forest succession (increase in shade tolerance), climate warming (increase in drought tolerance and temperature preference), and isolation by distance (e.g., forest fragmentation and isolation are linked to decreased seed mass).

## 2 STUDY AREA DESCRIPTION

Data were collected in Backus Woods, a 260 ha forest tract in Norfolk County, Ontario, Canada (42° 40 N and 80° 29 west), within the Lake Erie Lowland Ecoregion, also called the Southern Deciduous Forest Region or Carolinian Zone in Canada (Crins, Gray, Uhlig, & Wester, 2009; an area of about 24,000 km^2^). The tract occurs in the Central Hardwood Forest (Fralish, 2003; Fralish & Franklin, 2002), or part of the Beech‐Maple‐Basswood Forest Region (Dyer, 2006), and the climate is humid temperate with cold, snowy winters and hot, humid summers. The average annual temperature ranges from 6.3 to 9.4°C and varies from –9.6°C in January to 26.5°C in July, and the average annual precipitation is 776 to 1,018 mm (Ecoregions Working Group, 1989; Hewitt, 1998; Mackey, McKenney, Yang, McMahon, & Hutchinson, 1996b, 1996a). In Canada, the Carolinian Zone is dominated by agricultural and urban land with small pockets of mixed and deciduous forests (Elliott, 1998). Where forests are mature, *Acer saccharum*, *Fagus grandifolia*, *Quercus rubra*, *Q. alba*, *Carya ovata*, *Juglans nigra*, and *J. cinerea* are the main species. On moist sites, typical species include *Ulmus americana*, *Populus deltoides*, *P. balsamifera*, *Fraxinus pennsylvanica*, *F. nigra*, and *Acer saccharinum*. On drier sites, *Quercus velutina* and *Q. muehlenbergii* are more common. Species such as *Liriodendron tulipifera*, *Platanus occidentalis*, and *Carya cordiformis* are found on moist slopes (Environment Canada, 1996). Most of the original forested land cover in the Carolinian Zone was cleared over the past 200 years, and only about 14% of the region remains forested according to the Ontario Land Classification, SOLRIS (OMNRF, 2002). Prior to Euro‐American settlement, this landscape was predominantly forested but also contained patches of prairie, savanna, and marsh (Larson et al., 1999; Waldron, 2003).

The vestigial eolian dune formations at Backus Woods have formed 15–25 m high ridges interspersed with seasonally flooded lowlands, resulting in considerable topographic heterogeneity, and correspondingly diverse upland and lowland woody communities (Varga, 1985). Mean elevation in both years was 209.8 ± *SD* 2.8 m (range 193 m to 217 m—derived from digital elevation models for all grid points), and average slope was 5.1% ± *SD* 5.3 in 1985 and 5.4% ± 5.6 in 2009 (M. Woods, pers. comm.). Backus Woods harbors 543 vascular plants (21% of all plant species in the Province of Ontario), including tree species at risk (e.g., *C. dentata*, *C. florida*), nationally significant stands of large‐sized Carolinian tree species (*N. sylvatica* and *L. tulipifera*), forest‐dependent understory plants, and vertebrate species at risk (Varga, 1985). It is one of the best representative examples of the Southern Deciduous Forest in Canada with parts of the forest dating back to Euro‐American settlement (Richart & Hewitt, 2008), and 75% of it is considered “old‐growth” (Varga, 1985). Historically, a dominant foundation tree in Backus Woods, *C. dentata*, was ravaged by chestnut blight (*Cryphonectria parasitica*) beginning in the 1920s and it was subsequently replaced by *Quercus* spp. and *Acer* spp. Some selective logging occurred in the late 1800s and early 1900s, and the most recent logging was carried out between 1930 and 1966, mostly of *Quercus* species (Varga, 1985), perhaps affecting 25% of the forest; since 1966, there have been few interventions. In 1985, Backus Woods was established as a benchmark forest with two goals; first, maintaining the old‐growth structural characteristics of the tract in a landscape where most other remnants are selectively harvested, and second, maintaining its Carolinian species component.

## 3 MATERIALS AND METHODS

### 3.1 Sampling methods

**Figure 1:**
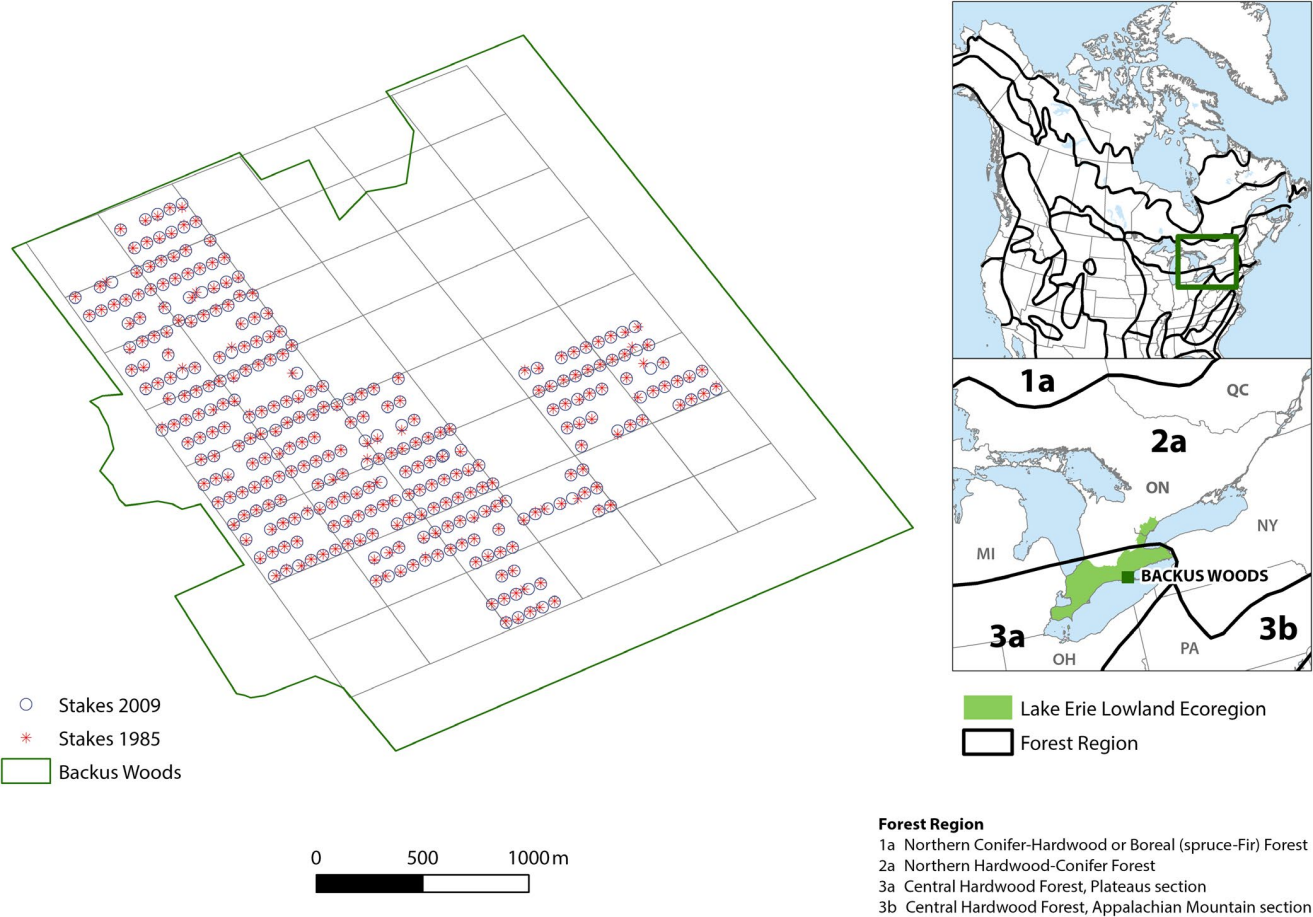
Map (right) showing location of Backus Woods within Forest Regions of North America and the Lake Erie Lowland Ecoregion in Ontario and inset fishnet grid overlay of cells at study site (left). Inset on right shows forest regions identified by Fralish and Franklin (2002) and Fralish (2003) for North America (modified map reproduced with permission from John Wiley & Sons, Inc.) Top inset on right shows all regions, lower inset those within southern Ontario. “Carolinian” tree species occupied ranges only within the Central Hardwood Forest, Plateaus, and Appalachian Mountain sections, 3a and 3b, respectively), approximately represented in Ontario by the Lake Erie Lowland Ecoregion); “central” species (range reached the Northern Hardwood‐Conifer Forest, Great Lakes Section, 2a); and “northern” species (ranges reached the Northern Conifer‐Hardwood or closed Boreal (spruce‐Fir) Forest,1a). Dark green square in Lake Erie Lowland Ecoregion on lower inset map denotes location of Backus Woods

A permanent grid of iron stakes was established by the Long Point Region Conservation Authority (LPRCA) in 1983; stakes were installed from east to west (bearing 240°) at 50 m intervals along a series of parallel rows, 100 m apart (Figure 1). Trees were first surveyed in 1985 at 528 of these permanent grid points (Varga, 1985). Because of time constraints and some difficulties in relocating stakes, only 397 grid points (totaling 3,176 sampled mature trees and saplings) could be resurveyed in 2009.

Each of the permanent grid points was used as a starting point to quantify tree species abundance using the point distance sampling method (Batcheler, 1971; Mitchell, 2010). Point distance methods involved first, dividing the area around each grid point into four quadrants in each compass direction. Within each of these quarters, the closest tree was located, identified to species, and the distance of the tree from the sampling point and its diameter at breast height measured (dbh 1.3 m). We treated mature trees (≥10 cm dbh) and saplings (≤9.9 cm dbh) separately in analyses for reasons previously mentioned. Some minor differences between years occurred in the locations used to initiate sampling. In 1985, surveyors tried to avoid disturbance to vegetation caused by the installation of stakes and so they began the point quarter distance sampling at a point away from the stake (mean distance 7.41 m). In 2009, the stake or grid point itself was used as the starting point for the point quarter distance sampling. To minimize variation due to these slight differences in vegetation sampling locations between years, we therefore examined the community as a whole by combining multiple stakes, not analyzing individual stakes. To do so, we constructed a fishnet overlay (cells of grid points) to group sampled grid points in ArcGIS (Version 10.22; ESRI, 2019). We optimized the location and the mesh size of the fishnet to contain the maximum number of grid points per cell with a minimum criterion of 10 grid points per cell (Figure 1). We used this optimized fishnet for both years so that despite slight variation in the location of sampled grid points (mostly 6 m offsets), grid points belonged to the same cell in both years. These new 20 cells, containing between 11 and 18 sampled grid points, were used as sample units in all subsequent analyses.

Mature trees and saplings were identified to species. Although data on tree seedlings were collected at each grid point, and seedlings have been used as indicators of change in forests (Kribel et al., 2011), we did not analyze our seedling data because of the large annual episodic fluctuations in seedling abundance. A total of 38 tree species were identified during the two surveys. We pooled the hybrid *Acer x freemanii* with *A. saccharinum* because we suspected observer bias; we also pooled *Fraxinus profunda* with *F. pennsylvanica* as the former was not recognized as a distinct species until 1992. Because we were also interested in analyzing changes in abundance of rare tree species, we had to account for potential biases in our sampling due to differences in grid point locations, and so eliminated extremely rare species that occurred at less than 1% of grid points. This selection retained 28 species. For each tree species, we measured temporal change between historical and recent surveys by computing species occurrence, relative abundance, and mean basal area by cells for each sampling period.

### 3.2 Species groups and functional traits

Tree species were assigned to three guilds based on their proportional range within the Lake Erie Lowland Ecoregion and according to their association with Forest Regions (Fralish, 2003; Fralish & Franklin, 2002). These were as follows: “Carolinian” tree species which had ranges only within the Central Hardwood Forest, Plateaus, and Appalachian Mountain sections (3a and 3b in Fralish & Franklin, 2002; Fralish, 2003, approximately represented in Ontario by the Lake Erie Lowland Ecoregion in Figure 1); “central” species with ranges that reached the Northern Hardwood‐Conifer Forest, Great Lakes Section (2a in Fralish, 2003; Fralish & Franklin, 2002); and “northern” species with ranges that reached the Northern Conifer‐Hardwood or closed Boreal (spruce‐Fir) Forest (1a in Fralish, 2003; Fralish & Franklin, 2002). To explore possible drivers of temporal community change, we compiled a suite of functional traits that act as indicators for environmental drivers (Mouillot et al., 2013): shade tolerance, to reveal successional processes; drought and waterlogging tolerance, as well as temperature preference, to highlight climate‐induced change; and seed mass to indicate isolation by dispersal limitation (Tamme et al., 2014; Appendix 1). Shade tolerance is expected to increase with succession and is often negatively correlated with other traits such as drought or waterlogging tolerance (Niinemets & Valladeres, 2006). Values for tolerance to shade, drought, and waterlogging were derived from Niinemets and Valladeres' (2006) and varied from 1 (very intolerant) to 5 (very tolerant). Seed masses were compiled from Hewitt (1998) and from the Seed Information Database (Royal Botanic Gardens, Kew, 2019).

Given trends demonstrating significant increasing mean annual temperatures in Norfolk County over the study period (Appendix 2), we analyzed temperature preferences for each tree species. We calculated this as the mean annual temperature across the species’ range, by combining climate and occurrence data (Devictor, Juillard, Couvet, & Jiguet, 2008). To do this, we overlaid interpolated climate data from the WorldClim database (mean annual temperature averages from 1970 to 2000 at a spatial resolution of 1 km^2^; Fick & Hijmans, 2017), and occurrence data from multiple forest inventory databases in eastern North America (collated for the “Quantifying and mapping impact of climate change on the forests productivity in eastern Canada” QUICC‐FOR project; https://github.com/QUICC‐FOR) for all tree species studied. Following Devictor et al. (2008), the mean annual temperature for each occurrence was extracted, and the mean of those temperature values used as an index of each species’ temperature preference (Appendix 1).

## 4 STATISTICAL AND DATA ANALYSIS

Because the cells contained varying numbers of sampled grid points (11–18), the results of significance tests could be influenced by variable sampling effort. To correct for this uneven sampling, we evaluated significance tests by subsampling grid points inside each cell (Burnham & Anderson, 2002) in all statistical analysis detailed in the following section. Subsampling is a resampling technique similar to bootstrapping. To do this, we (a) selected *n* = 10 grid points without replacement per cell from both surveys to create a subsample; then (b) computed tree species occurrence, relative abundance and mean basal area per cell; (c) performed the test; and (d) calculated the *p*‐value for the test. We repeated these four steps *r* = 1,000 times to obtain a distribution of *p*‐values (figures provided in Supplementary Appendices) and reported the mean *p*‐value in the results section. This procedure allowed us to include all of the grid points in the analyses, while removing the effect of uneven sampling and reducing the influence of any specific grid points due to small changes in location between years (see Kapfer et al., 2017 for a discussion of this problem).

### 4.1 Changes in mature trees and saplings at species and guild levels

To reveal structural and compositional changes in Backus Woods, we first examined temporal changes in individual species occurrence (presence/absence), relative abundance, and basal area. We then compared these measures between years using a Wilcoxon test for paired samples. Subsequent analyses for homogenization and functional traits were computed only on relative abundance data because we wanted to focus on change in composition related to tree recruitment and mortality, rather than change in basal area due to succession.

### 4.2 Evidence for homogenization and change in community composition of mature trees and saplings

We explored temporal change in β‐diversity (homogenization or differentiation) using a test for the homogeneity of multivariate dispersions (Anderson, Ellingsen, & McArdle, 2006). This analysis assessed the variability in species composition among communities during each sampling period. For each sampling period, we computed the site distance to centroids (*betadisper* function) based on Hellinger dissimilarity matrices (*n* x *n* sites), an appropriate distance measure for β‐diversity studies (Legendre & De Cáceres, 2013), and tested for change in β‐diversity using permutational ANOVA (9,999 permutation of residuals; *permutest* function). A decrease in the multivariate site distance to the time‐specific centroid is interpreted as biotic homogenization, while an increase indicated biotic differentiation.

To investigate changes in community composition over time, we performed a permutational multivariate analysis of variance (PERMANOVA; *adonis* function with 9,999 permutations), again using Hellinger distances (Anderson 2001, 2017). For visualization, we used Principle Coordinate Analysis ordination (PCoA) with Hellinger distances. Using these same two Hellinger dissimilarity matrices, we partitioned spatial β‐diversity for each sampling period into two components: Local Contributions to β‐diversity (LCBD, which is a measure of the uniqueness of sites in terms of their species composition), and Species Contributions to β‐diversity (SCBD, which is the amount of variation in individual species across the study area; Legendre & De Cáceres, 2013). In order to quantify changes in spatial β‐diversity through time and especially in terms of uniqueness, we computed the differences between contemporary and historical LCBD values, as LCBD_T2_ – LCBD_T1_.

### 4.3 Changes in functional traits in mature trees and saplings as indicators of drivers of change

We computed community indices for each trait (community weighted means of traits, CWM; Miller, Damschen, & Ives, 2019) as the mean of the species traits (temperature preference, tolerance to shade, waterlogging, drought, and log‐transformed seed mass index) weighted by the abundances of the species present in that cell. We tested for change in the mean trait value of the tree species community between sampling periods using a paired *t* test. We also calculated the species contribution to change in β‐diversity (SCBD) in community traits.

We carried out all statistical tests in R version 5.5.1 (R Development Core Team, 2016) using packages adespatial (Dray et al., 2020), FD (Laliberté & Legendre, 2010; Laliberté, Legendre, & Shipley, 2014), raster (Hijmans, 2020), sp (Pebesma & Bivand 2005), stats (R Development Core Team, 2016), and vegan (Oksanen et al. 2019).

## 5 RESULTS

### 5.1 Changes in mature trees and saplings at species and guild levels

Backus Woods was clearly dominated by the northern tree species group, and species in the central and especially Carolinian group were often comparatively rare, making statistical evaluation of changes in their occurrence and abundance challenging.

**Figure 2:**
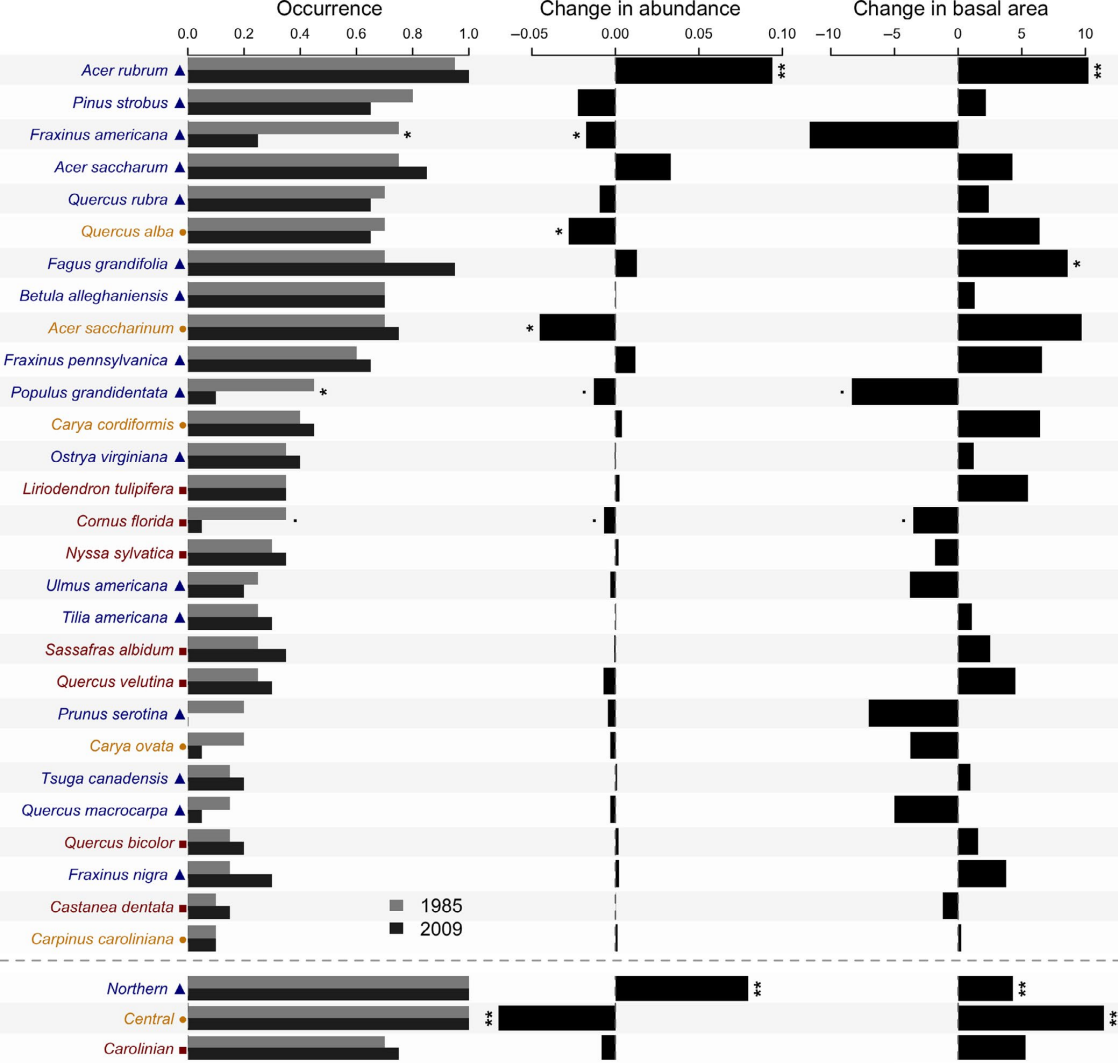
Change in species occurrence, abundance, and basal area of mature trees at Backus Woods between 1985 and 2009. “Occurrence” corresponds to percentage of cells occupied in each time period, while “change in abundance” and “change in basal area” corresponds to changes in mean values over all cells. Species groups indicated by color and symbols: northern species, blue triangle; central species, yellow circle; and Carolinian species, red square. Asterisks on all three panels represent significance levels (•*p* < .1; *p < .05; **p < .01) of bootstrapped paired Wilcoxon's tests comparing species between years. Results are shown by pooled species groups below main graph

**Figure 3:**
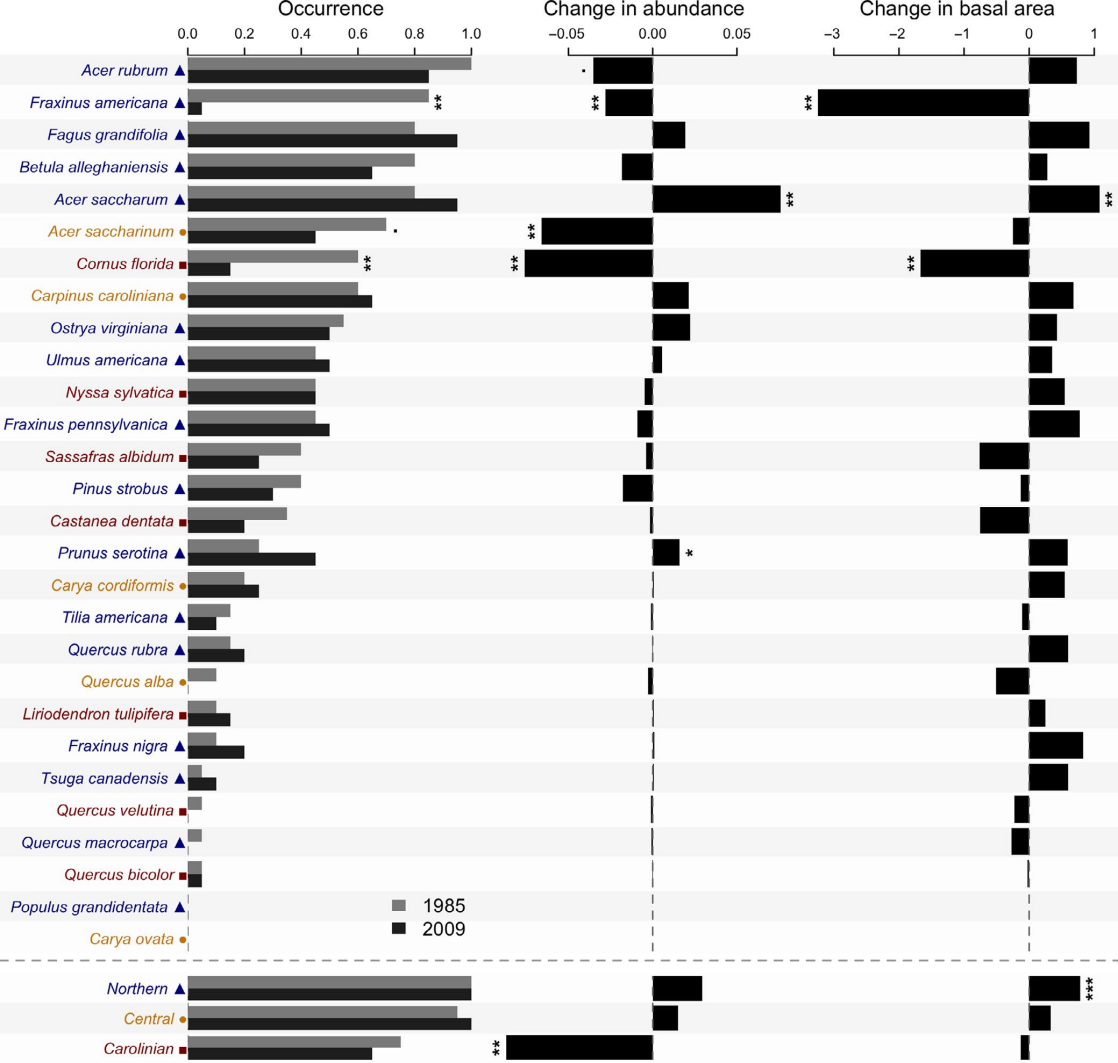
Change in species occurrence, abundance, and basal area of sapling trees at Backus Woods between 1985 and 2009. See Figure 2 for details

The top five mature tree species were all in the northern guild and in order of occurrence in the 20 cells were *A. rubrum*, *P. strobus*, *Fraxinus americana*, *Acer saccharum*, and *Q. rubra* (Figure 2). In terms of occurrence, two northern species (*F. americana* and *Populus grandidentata*) and one Carolinian species (*C. florida*) declined significantly between 1985 and 2009 (Figure 2). In the case of saplings, results were somewhat similar in terms of significant differences (Figure 3). For example, for occurrences, significant decreases were found for *F. americana* in the northern group, for *A. saccharinum* in the central group and *C. florida* in the Carolinian group.

More striking results were found for mature tree relative abundance. By far the most abundant species, *A. rubrum*, showed a highly significant increase between sampling periods (Figure 2). No other species increased significantly in relative abundance, but there were significant declines in two northern species (again *F. americana* and *P. grandidentata*), two central species (*Q. alba* and *A. saccharinum*), and one Carolinian species (*C. florida*). Most other Carolinian species showed little or no change but generally had low abundances (Figure 2).

We found significant increases in the overall abundance of the northern group for mature trees, while abundance of the central group declined significantly (Figure 2). For saplings, significant increases in relative abundance occurred in only one northern species (*A. saccharum*), whereas two species demonstrated significant decreases (including *F. americana*, and *P. serotina*) and one species had marginally significant decreases (*A. rubrum*). Both *A. saccharinum* in the central group and *C. florida* saplings in the Carolinian group also showed significant declines in relative abundance, as in mature trees. Although not significant individually, all seven Carolinian species either declined or showed no change in relative abundance (Figure 3). Strikingly, when pooled, the focal Carolinian group showed a significant decline in the overall abundance of saplings (Figure 3).

As expected, most mature tree species increased in basal area but significant increases occurred in only two species: *A. rubrum* and *Fagus grandifolia*. Significant declines occurred in *P. grandidentata* and *C. florida*. Furthermore, we found significant increases in basal area of the northern and central groups for mature trees (Figure 2). For sapling basal area, significant increases occurred for one northern species, *A. saccharum*, and significant decreases for another northern species, *F. americana* and one Carolinian species, *C. florida*. For the combined groups, there was a significant increase in the basal area of northern species’ saplings (Figure 3).

### 5.2 Evidence for homogenization and change in community composition of mature trees and saplings

**Figure 4:**
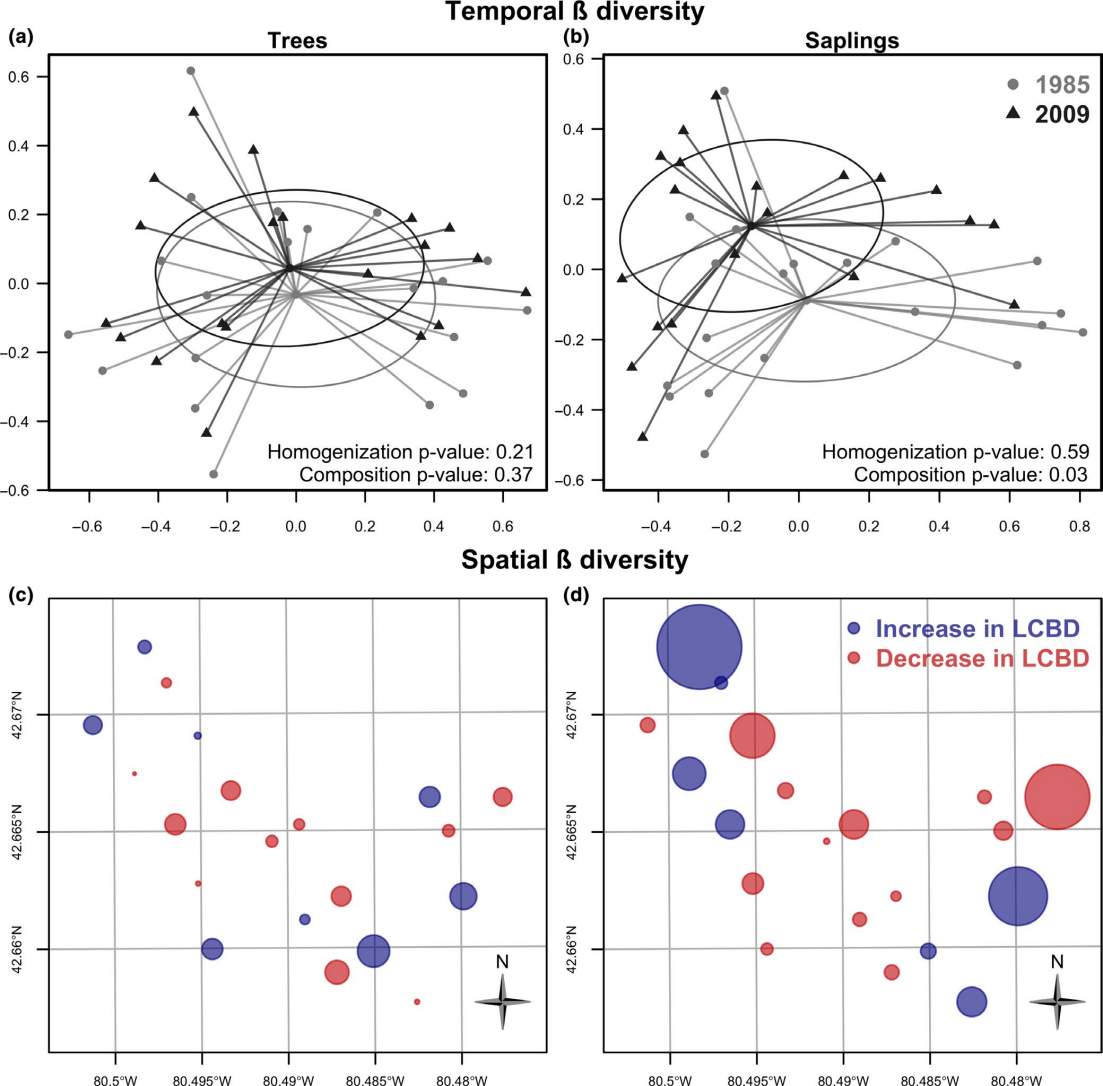
Principle coordinate analysis (PCoA) ordination showing compositional change in mature trees (a) and saplings (b) at Backus Woods between 1985 and 2009. Temporal β‐diversity was measured as the multivariate dispersion of cells around their centroid (based on the Hellinger distance) and was compared using tests for the bootstrapped multivariate homogeneity of group dispersions. Change in species composition was analyzed using bootstrapped permutational multivariate analysis of variance. Circles are ellipses of standard deviation; note the distinct shift in the ellipse for saplings reflecting changes in species composition. Bubble map of temporal change in local contribution to spatial ß‐diversity (LCBD) for trees (c) and saplings (d). Bubbles (sites) are proportional to the change in LCBD values (LCBDt2 – LCBDt1). The color represents the direction of change, where an increase in LCBD (increase in uniqueness of species composition) is in blue while a decrease in LCBD (decrease in uniqueness) is in red.

Despite the substantial increase in *A. rubrum*, we found no statistical evidence for homogenization of mature tree communities at Backus Woods (Figure 4a): There was no significant change in β‐diversity between 1985 and 2009 (*p* = .21). Similarly, no significant compositional change occurred between sampling periods (*p* = .37). Similar results were found for saplings in terms of homogenization/differentiation (*p* = .59; Figure 4b). However, a significant difference was found in sapling composition between 1985 and 2009 (*p* = .03), demonstrating that forest community structure is starting to alter (distributions of *p*‐values for homogenization/ composition are in Appendices S3 and S4).

The spatial patterns in local contributions to β‐diversity (LCBD, that is, increases and decreases in uniqueness of cells’ species composition over time) within Backus Woods were strikingly different between mature trees and saplings (Figure 4c,d). First, the magnitude of increases and decreases in LCBD were much greater in saplings than mature trees. Second, there was some evidence that decreases in sapling LCBD occurred more in the central part of the forest whereas increases occurred at the northern and southern edges. However, an exception was that sapling LCBD decreased markedly in the eastern part of the forest (42.665° N, 80.40° W). This area is in the lowland swamp (Dedrick Creek) part of the forest, and the effects in some cells may be due to declines in *A. saccharinum* (this was checked by examining spatial patterns for this species). Conversely, a large increase in LCBD occurred in the northwestern sections of the forest (42.67° N, 80.5° W) and to a lesser extent in the south‐east (42.66° N, 80.48° W; Figure 4d). For mature trees, the largest increases in LCBD were in the south‐east (e.g., 42.66° N, 80.48° W). As for saplings, spatial patterns suggested decreases in LCBD toward the center of the forest and increases at the edge (Figure 4c).

In terms of species contributions to β‐diversity (SCBD), the main changes in mature trees were attributable to decreased contributions of *A. saccharinum* and *Q. alba* (central), and *F. grandifolia* (northern) and increased contributions of *A. rubrum*, *A. saccharum*, and *Betula alleghaniensis* (Appendix 5). In saplings, the main changes were attributable to some of these same species (e.g., declines in contribution of *A. saccharinum* and *F. grandifolia*) but also *C. florida* (Appendix 6).

### 5.3 Changes in functional traits in mature trees and saplings as indicators of drivers of change

**Figure 5:**
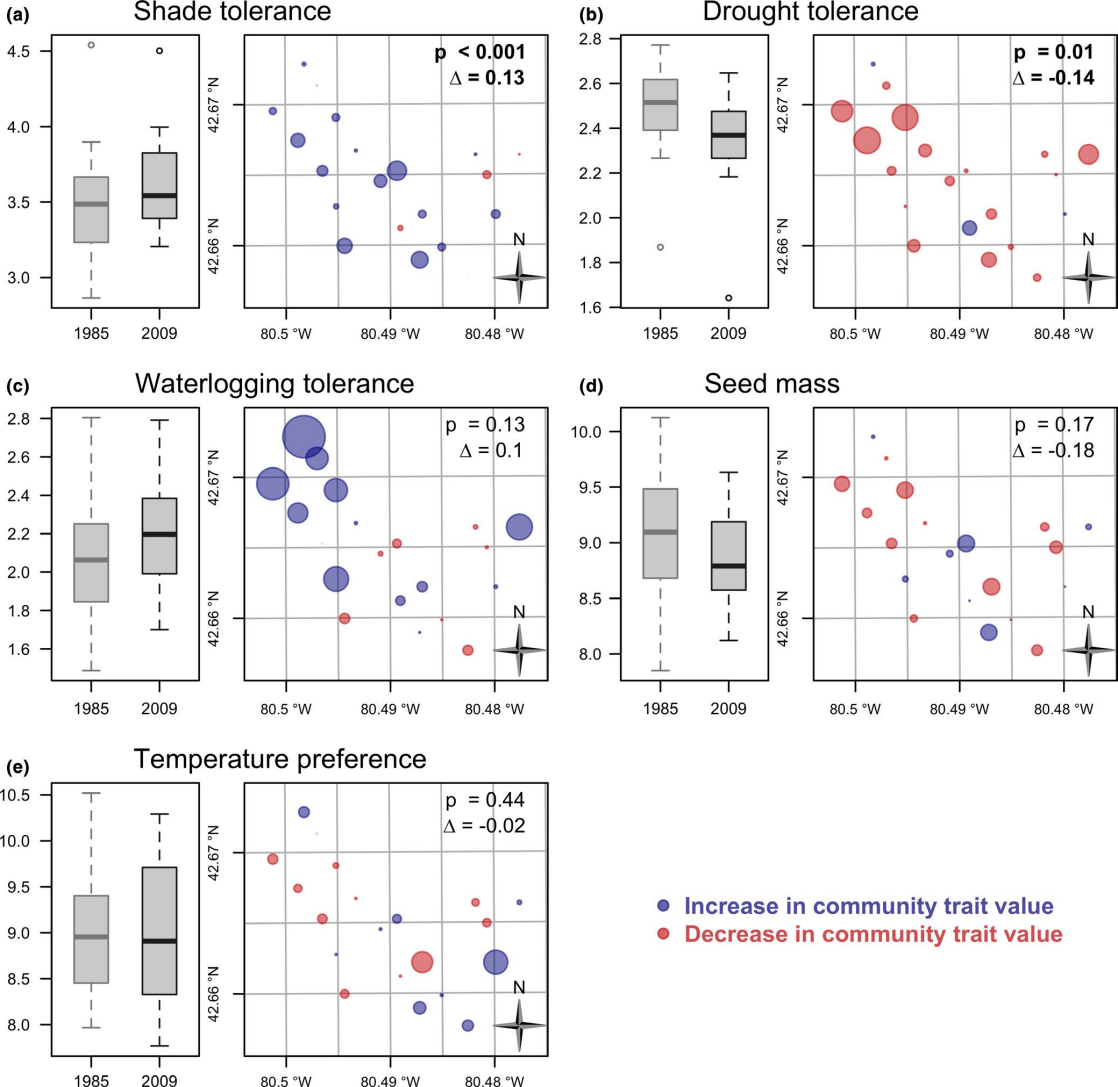
Temporal change in community trait values for mature trees. For each trait, boxplot on the left compares distribution (median and quartiles) of community weighted trait between 1985 and 2009, while the map on the right represents change in community weighted trait values for each grid point with bubbles proportional to their value. Community trait values that increased from 1985 to 2009 are shown in blue, and the ones that decreased are in red. *p*‐Values are from subsampled paired Student's *t* tests to evaluate change in mean trait values. Δ denotes change in community trait values

We observed a significant increase in average shade tolerance of mature tree species between the two sampling periods, and this increase was spread across the forest (*p* < .001; Figure 5a). Moreover, average drought tolerance of mature tree species decreased (*p* = .01; Figure 5b). The change in this trait revealed strong spatial patterns, with greater differences in the northern part of the forest (Figure 5b). Waterlogging tolerance showed increases, but the difference between time periods was not significant (Figure 5c). Average seed mass decreased, but the change was not significant (Figure 5d). Finally, no change occurred in the average temperature preference of mature tree species (Figure 5e).

**Figure 6:**
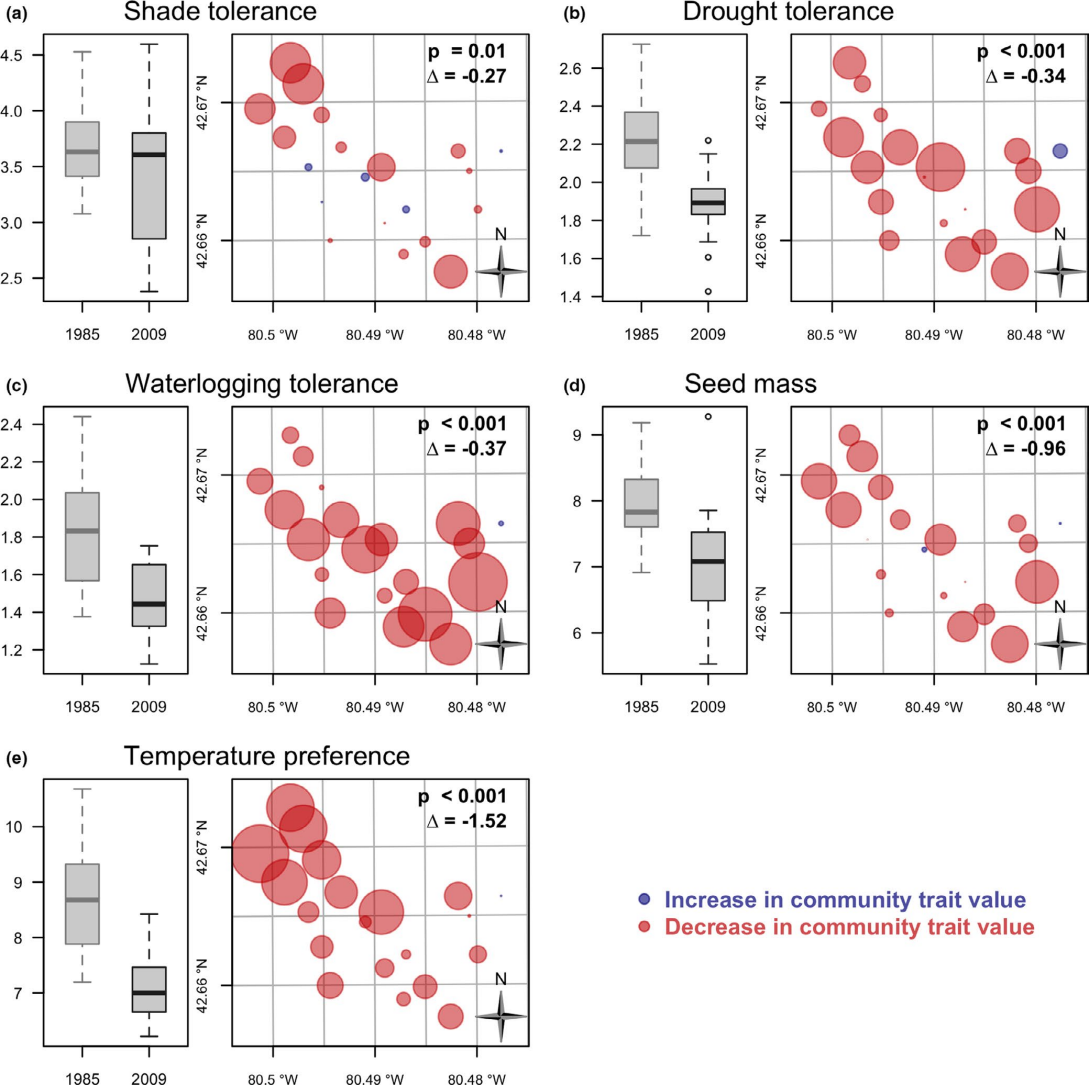
Temporal change in community trait values for saplings. See Figure 5 for details

In contrast to mature trees, for saplings, significant decreases were found for all traits (Figure 6a–e, *p* < .001 for all traits except shade tolerance where *p* = .01). Community trait values demonstrated declines scattered throughout the forest, though with some tendency for larger values for some traits at the edges and less change in the interior (Figure 6a,d). However, community values decreased markedly in the northwestern part of the forest for temperature (Figure 6e) and waterlogging tolerance in the south‐east (Figure 6c). Distributions of *p*‐values for trees and saplings are shown in Appendices S7 and S8, respectively. Analysis of species contributions to change in community traits demonstrated that for trees, the main changes in traits were driven by *A. rubrum* (increases), and to a much less extent, *A. saccharinum* and *Q. alba* (decreases; see Appendix 9). However, for saplings, species contributions to changes in traits were mainly due to decreases in *C. florida* and *A. saccharinum* and increases in *A. saccharum* (for shade tolerance, drought tolerance, and seed mass; Appendix 10). In terms of correlations between temporal changes in community traits (CWM at t_2_ – CWM at t_1_), results indicated that change in shade tolerance was negatively correlated with change in drought and waterlogging tolerance for mature trees but not for saplings (Appendix 11).

## 6 DISCUSSION

### 6.1 Overview

Our results demonstrated a striking difference in trends of mature trees and saplings between the two sampling periods. First, unlike mature trees, saplings showed significant changes in composition over time. However, no evidence was found for homogenization in either life stage during this study period. Second, analysis of saplings as a group revealed declines in abundance of Carolinian tree species, whereas in the case of mature trees, northern species increased and central species declined. Among northern species, our results demonstrated a dramatic and significant increase in the relative abundance of *A. rubrum*. Third, the magnitude of spatial changes in LCBD was much greater in saplings than mature trees and there was some evidence that the magnitude of changes (increases and decreases) occurred at the northwestern and southeastern edges of the forest, rather than the center. Fourth, a marked shift occurred in spatial patterns of β‐diversity in saplings mostly due to replacement; no such change occurred in mature trees (Appendix 12). Finally, analysis of community traits indicated that all traits declined significantly for saplings whereas there was a significant increase in shade tolerance and decline in drought tolerance for mature trees.

### 6.2 Absence of taxonomic homogenization and changes in sapling composition over time

Generally, models of forest dynamics predict that eastern North American temperate forests pass through a series of stages as they age—initially fast‐growing and light‐demanding (shade‐intolerant) species establish to be slowly replaced by slower‐growing, shade‐tolerant species that eventually form the forest canopy—unless this process is disrupted by disturbance (Lienard, Florescu, & Striegul, 2015). If disturbances are infrequent, then the late successional, shade‐tolerant species may eventually dominate the canopy and suppress other species; such old forests may be at temporary equilibrium (Runkle, 2013). This situation, where there is slow canopy turnover and extreme longevity of trees, was referred to as a “storage effect” by Chapman and McEwan (2016), and where canopy composition remains the same over hundreds of years despite environmental perturbations (thus, the storage effect is a mechanism to explain species’ coexistence; Chesson, 1985). Lack of disturbance can also cause convergence in community composition (i.e., low β‐diversity) by decreasing environmental filtering across environmental gradients (see Myers, Chase, Crandall, & Jiménez, 2015). Backus Woods appears to be following these same trends given the increasing proportion of more shade‐tolerant tree species.

As we suspected, the 24‐year interval between surveys and the lack of major disturbances appears to have been too short a period for detecting net change in β‐diversity for mature trees: Backus woods contains many old trees, and losses in some species are compensated by gains in others. Chapman and McEwan (2018) also found little compositional change over a 30‐year period in an old‐growth forest in Kentucky, but other studies of trees in mature forest have found significant changes over similar or slightly longer time periods ranging from 20 to 42 years (Chapman & McEwan, 2016; Lowney et al., 2016; Pinheiro et al., 2008; Runkle, 2013), perhaps related to disturbance events. However, at Backus Woods, the significant compositional change in the sapling layer between sampling periods suggests a trend toward increasing northern species with more shade tolerance. This may exacerbate the trend in decreasing numbers of less shadetolerant Carolinian species and continue to reduce their proportions in the future canopy (see Appendix 13 for size class changes over time, and Appendix 14 discussion). We also acknowledge the possibility that sampling biases may have caused some of the differences in sapling community composition over time, given that in 1985 disturbed areas at the stake were avoided during sampling and more regeneration at Backus Woods occurs in gaps (Bowles, 1998).

That compositional changes were evident in saplings supports findings elsewhere that young cohorts can be more sensitive indicators of environmental change than mature trees (e.g., (Kribel et al., 2011; Ramage et al., 2017; Schumacher & Carson, 2019). Mathys et al. (2018) reasoned that the fate of tree seedlings at the edges of their distributional ranges should be more sensitive indicators of climate change than mature trees. Supporting this, in Québec, northward range shifts in saplings of 16 tree species were greater in saplings than mature tree life stages (Sittaro et al., 2017). Other studies in temperate eastern North America have indicated little or no change in mature trees over time in old forests, but have found substantial changes in sapling and seedling species composition (Kribel et al., 2011). Our results from the partitioning of β‐diversity provided confirmation that local‐scale changes in saplings were mostly due to shifts in replacement (turnover; Appendix 12). Similarly, in temperate forests in Wisconsin, Wang et al., (2018) found that species replacement was the most important component of community dissimilarity and attributed this to the clustering of species at local scales in addition to dispersal limitation.

We expected to find homogenization in both the mature tree cohort but more especially in the sapling cohort, given the changes in composition and declines in CWM functional traits (see preceding and following sections). In central Pennsylvania, Schumacher and Carson, (2019) found that understorey sapling communities were a homogenized and smaller subset of canopy tree species (including loss of *Quercus* saplings) which may indicate that forest composition will change over time. In their study, most of the species concerned were shade and browse tolerant, and fire intolerant (e.g., *Acer* spp.). Although *A. rubrum* did not show increased abundance in the sapling cohort at Backus Woods, it did increase in its contribution to sapling SCBD and sapling *A. saccharum*, another shade‐tolerant species, also increased in abundance. However, other species, including two species affected by diseases (*C. florida* and *F. americana*) and one by hybridization (*A. saccharinum*), showed significant declines in saplings, suggesting that the contribution of these trees to future canopy and subcanopy (for *C. florida*) composition may be lower than historically and contribute to homogenization.

Our lack of support for homogenization at Backus Woods in spite of large increases in *A. rubrum* is somewhat similar to the findings of Chapman and McEwan, (2016) in Kentucky. In comparing forest trajectories in the Lilley Cornett Woods over a 30‐year period, they hypothesized that increased abundance of mesophytic species would lead to compositional homogenization in the understory, midstory, and upper canopy at their study site. However, they only found strong evidence for mesophication in the understory and not in the canopy. This was driven by decreased abundance of *Quercus* and *Carya*, but not due to increases in *Acer* species, since these were widespread and abundant in 1979, the first survey date. Moreover, in northern Wisconsin, no evidence of homogenization was found in less disturbed forests but pine barrens succeeding into closed canopy forests have become widely homogenized (Li, Poisot, Waller, & Baiser, 2018). At Backus Woods, lack of homogenization could be attributed to: a) the fact that *A. rubrum* was already abundant during the 1985 survey so that compositional differences were not great between sampling periods; b) the fact that other tree species were also increasing or decreasing, thus effectively compensating for the effect of increased dominance of *A. rubrum* or c) that topographic gradients and environmental filtering or community structural mechanisms override species compositional change. In relation to the last hypothesis c), strong topographic gradients at Backus Woods could disguise shifts in compositional similarity but environmental filtering may be difficult to disentangle from competition among species (Cadotte & Tucker, 2017).

Summarizing, the situation at Backus Woods may be somewhat different from the scenarios discussed by Olden and Poff, (2002), who found greatest homogenization where communities were invaded by similar species leading either to no extinction or differential extinction of native species. An analogy may be that *A. rubrum* could be seen as an invasive native species impacting the community at Backus Woods and potentially the rare Carolinian tree species. More generally, homogenization would be expected in forest fragments in anthropogenic landscapes such as Backus Woods, where at a local scale, “sensitive” species are extirpated, and “tolerant” species increase in fragments. For example, in fragmented rain forests in Mexico, disturbance‐adapted species increased in fragments but rare and shade‐tolerant species decreased (Arroyo‐Rodríguez et al., 2013).

Given its ability to grow in a wide range of soil and light conditions, early maturation, seed‐banking, and its expected increase with climate change (Taylor et al., 2017; Boisvert‐Marsh, Royer‐Tardiff, Nolet, Doyon, & Aubin,^2020^), we predict that *A. rubrum* will likely continue to exploit increasingly available gaps created by diseased trees (e.g., *Fraxinus* spp., *Fagus*, *Cornus*, and *Castanea*) at Backus Woods and replace other rare species, potentially resulting in future homogenization. Although the slight nonsignificant decline in *A. rubrum* saplings appears contrary to this prediction, these results are not unexpected given that *A. rubrum* is more light‐demanding, shorter‐lived and slightly favored by white‐tailed deer as browse compared to *A. saccharum* saplings (Bradshaw & Waller, 2016; Waller, Johnson, & Witt, 2017), which increased significantly. The important point is that the trend for *Acer* species to increase and dominate the future canopy may continue, thus eventually outcompeting Carolinian and central tree species.

### 6.3 Spatial changes in LCBD greater in saplings than mature trees

The spatial patterns in LCBD indicated which cells were most different from the multivariate centroids of all cells (those that were exceptionally different; Legendre & Condit, 2019) and provided important information on potential processes that structure communities. We found that the magnitude of spatial changes in LCBD was much greater in saplings than trees, which confirms our overall assessment of changes in species composition in saplings. Moreover, there was a tendency for the magnitude of increases and decreases in LCBD to occur more toward the edges of the study area (especially the northwestern and southeastern edges). Forest edges are likely to experience elevated light conditions as well as other microclimatic differences (e.g., increased drought conditions, higher carbon) compared to forest interiors (Harper et al., 2005; Smith, Hutyra, Reinmann, Marrs, & Thompson, 2018). Given that the main environmental drivers influencing recruitment of saplings in temperate forest are changes in canopy structure following damage (canopy height erosion) as well as other disturbances creating suitable light conditions (Senécal, Doyon, & Messier, 2018) and herbivory (Ruzicka, Groninger, & Zaczek, 2010), this makes sense from an ecological perspective. Not surprisingly, the cell with the most significant increase in LCBD of saplings at Backus Woods experienced a disturbance. This was in the north tract where tree mortality was elevated beginning in 2007 due to several years of drought and an outbreak of European Gypsy Moth, *Lymantria dispar*, an invasive species (LPRCA unpublished report).

### 6.4 Shade tolerance increased and drought tolerance decreased for mature trees while declines occurred in all community traits for saplings

At a broad level, our findings suggest that temporal change in functional traits was more dramatic than changes in compositional dissimilarity, especially for the sapling cohort. In mature trees, significant increases occurred in shade tolerance and, as expected, drought tolerance was negatively correlated with shade tolerance and declined significantly. That shade tolerance was so important is not surprising given the overriding importance of this trait in forest dynamics (Valladares & Niinemets, 2008). Continued future shifts toward decreases in drought tolerance could compromise the resilience of the forest to adapt to changes in moisture regime, particularly extreme drought. This is because drought‐adapted species such as *Quercus* spp. may disappear from the forest and therefore not provide seed to the canopy gaps produced by overstory mortality driven by drought. Susceptibility to drought stress is believed to be greater in mature and larger trees because of their height and limited capacity to move water from their roots to canopy leaves (Bennett, McDowell, Allen, & Anderson‐Teixeira, 2015). Given that drought is one of the most pervasive biotic drivers of change in eastern North American forests (D’Orangeville et al., 2018; Pederson et al., 2014), and that it has been suggested that large episodic drought events play a role in disturbance trajectories (Pederson et al., 2014), this is an important finding. However, changes in these traits may also be a function of traits being intercorrelated; shade tolerance was negatively correlated with the two moisture‐related traits—drought tolerance and waterlogging tolerance (Appendix 11).

Overall, our analysis of species changes in community traits demonstrated that the main temporal differences were driven by increases in *A. rubrum* and to a much lesser extent, declines in *A. saccharinum* and *Q. alba* (Appendix 9). For example, *A. rubrum* has high tolerance to waterlogging (ranked fifth) suggesting that the significant increases in this trait over time are likely due to increased abundance of this species, rather than implying that the forest has become wetter. While many parts of Backus Woods are subject to seasonal flooding (e.g., the *A. saccharinum* swamps) and increases in species resistant to flooding (e.g., *A. rubrum*) could have been caused by alterations to flooding regime or adjacent lake levels, recent efforts to diminish the influence of tile drainage from adjacent agricultural lands on Backus Woods suggest that this is unlikely (personal observations). The contribution of mature *A. rubrum* to changes in traits is important ecologically because this species may influence forest hydrology and nutrient availability. In Kentucky, Alexander and Arthur (2010) found that expanding *A. rubrum* could impact spatial distribution of water and nutrients via stemflow and thereby increase its competitive success.

In contrast to mature trees, in saplings species contributions to changes in traits were mainly due to decreases in *C. florida* and *A. saccharinum* (e.g., shade tolerance, drought tolerance, seed mass) and increases in *A. saccharum* for shade tolerance and seed mass (Appendix 10). Both *C. florida* and *A. saccharinum* showed strong declines in SCBD (Appendix 6). The dogwood anthracnose fungus (*Discula destructiva*), which arrived in southern Ontario during the 1990s, is the primary cause of widespread declines in the endangered *C. florida* throughout its North American range (Bickerton & Thompson‐Black, 2010). However, it has been suggested that increasing shade from a closing canopy and drought, two of the traits we examined, could render the species more susceptible to anthracnose and could also be a threat even in the absence of the disease (McEwan, Muller, Arthur, & Housman, 2000). In the case of *A. saccharinum*, this species had high waterlogging tolerance and both mature trees and sapling abundance declined significantly over our study period. While declines in *A. saccharinum* may be attributable to hybridization and/or competition with *A. rubrum*, the repercussions of these declines could be that fewer tree individuals are present that are tolerant of waterlogging, something that could increase with climatic events (e.g., increased intensity of seasonal flooding). Thus, our results provide further evidence for the fact that soil moisture plays a key role in forest dynamics (see Pederson et al., 2014). Closed canopy cover and the lack of disturbance in Backus Woods may explain why we did not detect any differences in temperature preference between time periods (De Frenne et al., 2013), despite average temperatures increasing at a regional level in southern Ontario (Appendix 2). Although thermophilization (increases in warm‐adapted species) is believed to be occurring in forests of North America, it has been associated with disturbance which opens the forest canopy and creates conditions for species with higher temperature tolerances (Brice, Cazelles, Legendre & Fortin, 2019). Among other traits, we expected to find differences in dispersal characteristics in mature trees since species in forest fragments are more often wind‐dispersed and we expected a decline in tree species with heavier seeds or those with animal‐dispersed seeds (McEuen & Curran, 2004); however, no significant effect of sampling period was found. We speculate that this is most likely because the analysis for this trait was carried out over too short a time period; the local population at Backus Woods may be maintained by short‐distance dispersal.

## 7 CONCLUSION

The declines in mature life stages in the central group and increases in the northern group provide further support for the widespread regional phenomenon of “mesophication” of forests in eastern North America (Chapman & McEwan, 2016; Nowacki & Abrams, 2008) but has not been previously documented in the published literature for southern Ontario. This mesophication involves a switch from *Quercus* species to *Acer* species, especially *A. rubrum* but also *A. saccharum* (Chapman & McEwan, 2016; Nowacki & Abrams, 2008; Rogers et al., 2008). In particular, we documented dramatic increases in *A. rubrum*. The change in this species had a large influence on the community trait analyses suggesting that it is influencing the functional forest composition and could potentially lead to future functional homogenization. Our study also illustrated that sapling composition can be a more sensitive indicator of environmental change than mature trees, and this is particularly so for legacy studies conducted over relatively shorter time periods. Despite warming temperatures (Appendix 2) predicted to facilitate continued existence or expansion of Carolinian tree species (Desprez et al., 2014), we found that Carolinian saplings declined as a group—supporting the second of our alternate hypotheses. We posit that this is because of the lack of disturbances needed for recruitment as well as a loss of the appropriate environmental conditions required—with flooding, drought, and light being the proximate factors. Because survivorship of different cohorts and species may alter over time (Boerner & Brinkman, 1996), it is uncertain whether the 2009 sapling composition at Backus Woods will be reflected in the future forest canopy. Nevertheless, the finding that community dissimilarity in saplings is shifting and that functional traits are declining in this ontogenetic life stage may require further investigation.

Increasing concern is being expressed about the resilience of northeastern forests to climate change (Rustad et al., 2012; Waller, Ash, Paulson, & Sonnier, 2015) and studies such as this one provide invaluable information on potential future compositional changes. It is possible that the continued existence of Carolinian tree species at the northern limit of their ranges has been facilitated by a combination of factors, including a lack of recent major disturbances and historical legacies (Waldron, 2003), often as a result of protecting mature trees through the network of parks and conservation land holdings in the Carolinian zone. While land managers may wish to consider restoration techniques to facilitate regeneration and succession for some Carolinian and central tree species to maintain species diversity (see Morelli et al., 2016; Morellii & Millar, 2018 regarding climate refugia forests), it is important that this is balanced with the primary management goal of maintaining the old‐growth characteristics of the forest. Restoration that encourages regeneration and succession of the less common species can form part of plans for managed forests and may occasionally match the objectives of certain protected areas, but not necessarily Backus Woods, where the protection of old‐growth characteristics may be a higher priority.

## CONFLICT OF INTEREST

None declared.

## AUTHOR CONTRIBUTION

**David Anthony Kirk:** Conceptualization (lead); Data curation (equal); Formal analysis (supporting); Investigation (equal); Methodology (equal); Project administration (lead); Supervision (lead); Writing‐original draft (lead); Writing‐review & editing (lead). **Marie‐Hélène Brice:** Formal analysis (lead); Methodology (equal); Software (lead); Visualization (lead); Writing‐review & editing (supporting). **Michael S. Bradstreet:** Conceptualization (equal); Data curation (equal); Funding acquisition (lead); Investigation (equal); Project administration (supporting); Resources (lead); Supervision (supporting); Writing‐review & editing (supporting). **Ken A. Elliott:** Conceptualization (supporting); Investigation (supporting); Supervision (supporting); Writing‐review & editing (supporting).

## Data Availability

Data available from the Dryad Digital Repository https://doi.org/10.5061/dryad.0gb5mkkzt (Kirk et al., 2020).

## Appendix 1

Tree species composition of Carolinian, central and northern groups and values for trait‐based functional groups (mean ± *SD*). Carolinian species (range only in Central Hardwood Forest, Plateaus, and Appalachian Mountains sections, 3a and 3b, respectively, in Fralish & Franklin, 2002; Fralish et al., 2003; *n* = 7), central species (range also in Northern Hardwood‐Conifer Forest, Great Lakes section, 2a in Fralish & Franklin, 2002; Fralish et al., 2003; *n* = 5), and northern species (range reached Northern Conifer‐Hardwood or closed Boreal (spruce‐Fir) Forest, 1a in Fralish & Franklin, 2002; Fralish et al., 2003; *n* = 16).

**Table:**

Group and species name	Acronym	Shade tolerance	Drought tolerance	Waterlogging tolerance	Seed mass^a^	Temperature preference
Carolinian						
*Castanea dentata*	CASDENT	3.06	3	1	12.75	9.66
*Cornus florida*	CORFLOR	4.87	2.92	1.1	9.22	15.28
*Liriodendron tulipifera*	LIRTULI	2.07	2.6	1.3	8.08	13.94
*Nyssa sylvatica*	NYSSYLV	3.52	2	1.87	10.97	14.62
*Platanus occidentalis*	PLATOCC	2.86	2.25	2.71	5.39	13.90
*Quercus velutina*	QUEVELU	2.72	3	1.07	12.64	12.34
*Sassafras albidum*	SASALBI	1.68	5	1.11	9.12	12.34
Mean		2.97	2.97	1.45	9.74	13.03
*SD*		1.04	0.98	0.63	2.62	2.04
Central						
*Acer saccharinum*	ACESACN	3.6	2.88	3.37	10.39	9.97
*Carpinus caroliniana*	CARCARO	4.58	2.02	2.3	7.31	15.9
*Carya cordiformis*	CARCORD	2.07	4	2.5	12.58	11.06
*Carya ovata*	CAROVAT	3.4	3	1.38	13.02	11.81
*Quercus alba*	QUEALBA	2.85	3.56	1.43	12.62	12.95
Mean		3.30	3.09	2.20	11.18	12.93
*SD*		0.93	0.75	0.83	2.40	2.13
Northern						
*Acer rubrum*	ACERUBR	3.44	1.84	3.08	7.59	9.28
*Acer saccharum*	ACESACC	4.76	2.25	1.09	8.91	6.93
*Betula alleghaniensis*	BETALLE	3.17	3	2	4.61	4.49
*Fagus grandifolia*	FAGGRAN	4.75	1.5	1.5	10.25	8.46
*Fraxinus americana*	FRAAMER	2.46	2.38	2.59	8.41	9.54
*Fraxinus nigra*	FRANIGR	2.96	2	3.5	8.63	4.92
*Fraxinus pennsylvanica*	FRAPENN	3.11	3.85	2.98	8.25	11.86
*Ostrya virginiana*	OSTVIRG	4.58	3.25	1.07	7.31	8.91
*Quercus macrocarpa*	QUEMACR	2.71	3.85	1.82	13.31	6.72
*Quercus rubra*	QUERUBR	2.75	2.88	1.12	12.46	9.67
*Pinus strobus*	PINSTRO	3.21	2.29	1.03	7.44	6.85
*Populus grandidentata*	POPGRAN	1.21	2.5	2	2.71	6.14
*Prunus serotina*	PRUSERO	2.46	3.02	1.06	9.15	4.69
*Tilia americana*	TILAMER	3.98	2.88	1.26	9.01	5.34
*Tsuga canadensis*	TSUCANA	4.83	1	1.25	5.49	6.87
*Ulmus americana*	ULMAMER	3.14	2.92	2.46	6.51	10.67
Mean		3.35	2.59	1.86	8.13	7.58
*SD*		1.01	0.78	0.83	2.65	2.25

^a^ Seed mass is the logarithm of the mass (mg) of 100 seeds.

## Appendix 2

Trends in mean annual temperature in Norfolk County, southern Ontario, including the study period (indicated by black triangles on *x*‐axis). Regression line is based on climate data from 1970 to 2010 which were extracted using a 2 km^2^ (60 arc sec) resolution grid in the ANUSPLIN climate modeling software (http://cfs.nrcan.gc.ca/projects/3/8; McKenney et al., 2011).

## Appendix 3

Distributions of *p*‐values obtained by subsampling multivariate tests for the homogeneity of variance (homogenization) and permutational multivariate analysis of variance tests (change in composition) for trees.

## Appendix 4

Distributions of *p*‐values obtained by subsampling multivariate tests for the homogeneity of variance (homogenization) and permutational multivariate analysis of variance tests (change in composition) for saplings.

## Appendix 5

Change in species contributions to β‐diversity (SCBD) between 1985 and 2009 for mature trees. Left panel shows values for 1985, centre panel for 2009, and right panel shows difference between years.

## Appendix 6

Change in species contributions to β‐diversity (SCBD) between 1985 and 2009 for saplings. Left panel shows values for 1985, centre panel for 2009, and right panel shows difference between years.

## Appendix 7

Distributions of *p*‐values obtained by subsampling paired sample *t* tests evaluating temporal change in CWMs for trees.

## Appendix 8

Distributions of *p*‐values obtained by subsampling paired sample *t* tests evaluating temporal change in CWMs for saplings.

## Appendix 9

Changes in species contributions to community traits in mature trees. Species contributions were assessed for each species by (a) replacing the abundance at *t*_2_ by its previous abundance at *t*_1_, as if the species abundance had not changed over time; (b) computing a new CWM’ *t*_2_; (c) then calculating ΔCWM’ using CWM’ *t*_2_ and CWM *t*_1_ as above; and finally (d) measuring the difference between ΔCWM’ and ΔCWM at each cell. A positive value indicated that the change in a given species’ abundance increased community trait values in a cell. Then, we determined the role of species gains and losses in ΔCTI by averaging their contributions for grid points where they increased and where they decreased. Acronyms for tree species are in Appendix 1.

## Appendix 10

Changes in species contributions to community traits in sapling trees.

See caption to Appendix 9. Acronyms for tree species are in Appendix 1.

## Appendix 11

Correlations between traits for mature trees (a) and saplings (b). Positive correlations are shown in blue shades and negative correlations in pink shades. Axes show Pearson's correlations on a scale of 1 to −1.

(a)

(b)

## Appendix 12

Triangle plots partitioning out spatial β‐diversity by sampling period (1985 and 2009). These show the contributions of three mechanisms—differences in abundance, replacement and similarity for mature trees (A and B) and saplings (C and D) in each sampling period (1985 and 2009). To decompose β‐diversity into temporal and spatial components, we focused on species replacement and richness difference or nestedness following Legendre (2014). Replacement refers to the “simultaneous gain and loss of species due to environmental filtering, competition, and historical events” or turnover if measured along spatial or environmental gradients.

We based decomposition of *temporal and spatial* β‐diversity on abundance data using the Ružička dissimilarity measure:

(B + C/(A + B + C), equivalent to Jaccard).

Abundance difference = |B − C|/(A + B + C);

Replacement = 2 × min(B,C)/(A + B + C);

Similarity = A/(A + B + C).

## Appendix 13

Size‐class distribution of trees and saplings for the three geographical species’ groups. Triangles on the *x*‐axis show the mean for each year (1985 and 2009).

## Appendix 14

### SUPPLEMENTARY DISCUSSION

Related to the importance of climate traits at Backus Woods, it has recently been postulated that a few rare climatic events in the late 1770s caused drought‐induced canopy mortality in eastern deciduous forests (Pederson et al., 2014). Interestingly, this coincides with the purported origin of Backus Woods (Larson et al., 1999). It could possibly explain how large *L. tulipifera*, which require large gaps to succeed and reach the canopy (Busing, 1995), still form a component of the mature tree canopy at our study site, although most are unlikely to be 230 years old. However, while early settlers may have discovered a forest partially established through the 1,770 drought event, most of the overstory at Backus Woods is believed to have originated from the heavy period of harvesting and land clearing that utilized fire in the county (Wilson, 2017; see also Suffling et al., 2003). Based on 77 trees cored in 2005, eight trees, mostly *Quercus* spp., were >120 years old and established between 1853 and 1881 and one tree in 1836 (K. A. Elliott, personal observations). The next cohort of seven trees (again mostly *Quercus* spp.) were 100–119 years old (established between 1886 and 1902). Younger trees were mostly *Acer* spp. and *Fraxinus* spp. The size class distribution for saplings and mature trees in each of the three groups is shown in Appendix 13. For the size‐class distributions of the Carolinian species, we expected an even‐aged distribution (unimodal) as these tree species tend to be shade intolerant or midtolerant and many would have become established at the same time.
